# Chronic leucine supplementation improves glycemic control in etiologically distinct mouse models of obesity and diabetes mellitus

**DOI:** 10.1186/1743-7075-7-57

**Published:** 2010-07-12

**Authors:** Kaiying Guo, Yi-Hao Yu, Jue Hou, Yiying Zhang

**Affiliations:** 1 Department of Pediatrics, Division of Molecular Genetics, Columbia University, New York, USA; 2 Department of Medicine, Division of Preventive Medicine, Columbia University, New York, USA; 3 Naomi Berrie Diabetes Center, Columbia University, New York, USA

## Abstract

**Background:**

Leucine may function as a signaling molecule to regulate metabolism. We have previously shown that dietary leucine supplementation significantly improves glucose and energy metabolism in diet-induced obese mice, suggesting that leucine supplementation could potentially be a useful adjuvant therapy for obesity and type 2 diabetes. Since the underlying cause for obesity and type 2 diabetes is multifold, we further investigated metabolic effects of leucine supplementation in obese/diabetes mouse models with different etiologies, and explored the underlying molecular mechanisms.

**Methods:**

Leucine supplementation was carried out in NONcNZO10/LtJ (RCS10) - a polygenic model predisposed to beta cell failure and type 2 diabetes, and in B6.Cg-A^y^/J (*A*^*y*^) - a monogenic model for impaired central melanocortin receptor signaling, obesity, and severe insulin resistance. Mice in the treatment group received the drinking water containing 1.5% leucine for up to 8 months; control mice received the tap water. Body weight, body composition, blood HbA1c levels, and plasma glucose and insulin levels were monitored throughout and/or at the end of the study period. Indirect calorimetry, skeletal muscle gene expression, and adipose tissue inflammation were also assessed in *A*^*y *^mice.

**Results:**

Leucine supplementation significantly reduced HbA1c levels throughout the study period in both RCS10 and *A*^*y *^mice. However, the treatment had no long term effect on body weight or adiposity. The improvement in glycemic control was associated with an increased insulin response to food challenge in RCS10 mice and decreased plasma insulin levels in *A*^*y *^mice. In leucine-treated *A*^*y *^mice, energy expenditure was increased by ~10% (p < 0.05) in both dark and light cycles while the physical activity level was unchanged. The expression levels of UCP3, CrAT, PPAR-alpha, and NRF-1, which are known to regulate mitochondrial oxidative function, were significantly increased in the soleus muscle of leucine-treated A^y ^mice whereas the expression levels of MCP-1 and TNF-alpha and macrophage infiltration in adipose tissue were significantly reduced.

**Conclusions:**

Chronic leucine supplementation significantly improves glycemic control in multiple mouse models of obesity and diabetes with distinct etiologies. The metabolic benefits of leucine supplementation are likely mediated via multiple mechanisms in different tissues, but are not necessarily dependent of weight reduction.

## Background

Impaired glucose metabolism and type 2 diabetes are prevalent metabolic disorders, and are commonly associated with obesity. Considerable interest has been generated in recent years in dietary approaches for the prevention and treatment of obesity and the associated insulin resistance and diabetes mellitus because the interaction between diet and genetic predisposition plays a significant role in the development of these metabolic disorders. In obese and insulin resistant individuals, protein-rich diets are associated with better glycemic control and plasma lipid profile, and, when used therapeutically for weight reduction, promote energy expenditure and greater relative fat reduction, compared to isocaloric, high carbohydrate or high fat diets [1-5]. However, the molecular mechanism for the observed metabolic benefits of protein-rich diet is not fully understood. It has been postulated that increased intake of leucine, an essential branched-chain amino acid (BCAA) and a natural component of dietary proteins, may play an important role in mediating the metabolic benefits of protein-rich diet [6,7]. Indeed, increasing evidence suggests that altered leucine/BCAA intake and metabolism could have significant effects on macromolecule and energy metabolism. Genetic knockout of branched-chain aminotransferase 2 (BCATm), which catalyzes the first step of BCAA catabolism, leads to dramatically elevated plasma levels of BCAAs, increased energy expenditure, and lean phenotype in mice [8]. Leucine supplementation with 50% food restriction results in lower adiposity in rats, compared to the control animals that are subjected to the same 50% food restriction without leucine supplementation [9]. Chronic supplementation with BCAAs also increases hepatic and muscle glycogen concentration in exercised rats [10]. However, metabolic effects of leucine and/or BCAA supplementation may be complex, and some of the beneficial effects have not always been seen. Newgard et al reported that dietary supplementation of BCAA reduces high fat diet-induced weight gain in mice, but induces insulin resistance [11].

We have investigated whether dietary leucine supplementation is able to mimic the effects of protein-rich diet on glucose and energy metabolism in C57BL/6J mice on a high fat diet (DIO mice) [7]. We have shown that doubling dietary leucine intake over a 14-week period significantly increases energy expenditure, attenuates high fat diet-induced weight gain, and improves glucose and cholesterol metabolism in these DIO mice [7]. However, given the complexity of the underlying causes for obesity and type 2 diabetes and of the potential effects of leucine and/or BCAA on energy and glucose metabolism [7-9,11,12], we sought in this study to further investigate the metabolic effects of long term leucine supplementation in two additional mouse models of obesity and diabetes with distinct etiologies and disease severities, and to explore the underlying molecular mechanisms. NONcNZO10/LtJ (RCS10), a congenic strain generated by combining quantitative trait loci from New Zealand Obese (NZO/HlLt) and nonobese nondiabetic (NON/LtJ) mice, is a recently established polygenic mouse model of obesity and type 2 diabetes [13]. Although the exact nature of the quantitative traits loci responsible for the obesity and diabetes phenotype of RCS10 is not yet fully defined, the polygenic nature and the relatively mild obesity of this model closely resemble human type 2 diabetes. Male RCS10 mice are characterized by maturity-onset obesity, hepatic insulin resistance, beta cell failure, and full-fledged diabetes around 6 months of age if they are put on a diet containing ~20% calories from fat [13,14]. The second model studied is B6.Cg-*A*^*y*^/J (*A*^*y*^), which segregates for a mutation (*yellow*) in the agouti gene that impairs melanocortin receptor signaling (MC3R and MC4R) in the central nervous system [15]. In the C57BL/6J inbred background, male *A*^*y *^mice are mildly hyperphagic, hypometabolic, and extremely insulin resistant. Although male *A*^*y *^mice are glucose intolerant, they rarely develop frank diabetes due to strong beta-cell compensations. Thus, the *A*^*y *^model, compared to the RCS10 model, represents the other end of the spectrum of impaired glucose homeostasis, in which insulin resistance dominates the disease process.

## Materials and Methods

### Animal husbandry, diets and leucine supplementation

Seven to eight-week-old male NONcNZO10/LtJ (RCS10) and B6.Cg-*A*^*y*^/J (*A*^*y*^) mice were purchased from Jackson Laboratories. Animal protocols were in compliance with the accepted standards of animal care, and were approved by the Columbia University Institutional Animal Care and Use Committee. Mice were maintained at 22°C on a 12:12 light-dark cycle (0700-1900), and had *ad libitum *access to the breeder chow (Purina Mouse Diet 20 5058, 21.8%, 21.6% and 56.6% calories from protein, fat, and carbohydrates, respectively). The breeder chow diet, which contains twice as much fat calories as the regular chow, increases the rate of weight gain in *A*^*y *^mice and is necessary for the development of overt diabetes with high frequency in male RCS10 mice (Jackson Laboratory website). Leucine was supplemented via the drinking water containing 1.5% (wt vol^-1^) L-leucine (Sigma, St. Louis, MO) as previously described [7,16]. Since rodents consume the majority of water with meals during the dark cycle, supplementation via drink water should achieve similar effects as supplementation through food. This method of supplementation was chosen mainly out of the consideration of convenience. The controls were sex- and age-matched mice fed the same diet with regular tap water as drinking water.

### Determination of body composition, plasma amino acid concentrations, and blood HbA1c, glucose and insulin levels

Body composition was determined using a Minispec TD-NMR Spectrometer (Bruker Optics, TX). HbA1c levels were determined using DCA 2000 Hemoglobin A1c (HbA1c) Reagent Kit (Bayer, HealthCare, LLC, Elkhart, IN). Plasma amino acid concentrations were determined by the Hormone Analytic Core of the Mouse Metabolic Phenotyping Center at the Vanderbilt University. Blood glucose levels were measured in tail-vein blood using Glucometer Elite (Bayer, Elkhart, IN). Plasma insulin levels were determined using an ELISA kit (Mercodia Inc, Winston Salem, NC). Because plasma glucose and insulin levels are regulated by different mechanisms in different feeding states, we measured them in three feeding states to assess the effect of leucine treatment on these mechanisms. The three feeding states are defined as: the fed state - measurements were taken at the 4^th ^hour of the dark cycle; the basal state - measurements were taken at the 7^th ^hour of the light cycle after 5 hour food and leucine deprivation; the fast state - measurements were taken after 24 hour food and leucine deprivation. During the food and leucine deprivation, all mice were supplied with the regular tap water to prevent them from dehydration. In order to minimize the effects of feeding manipulation and handling (collecting ~50 ul blood for insulin analysis) on their metabolism, the mice were tested in the three feeding states in the following order, the fed state, the basal state, and the fast state during the testing period after 2, 4 or 8 month leucine treatment. A 2-day resting period was given between testing, and the animals were allowed to recover from the 24 hour fast for at least one week. The interpretations of the data obtained in the different feeding states are described in the text where they are relevant. To further compare the regulation of glucose-insulin homeostasis in response to acute meal ingestion in leucine-treated and control mice, we also conducted a fasting-refeeding experiment in RCS10 mice at the end of 8 month leucine treatment. The mice in both leucine and control groups were deprived of food and leucine (for the leucine group) and supplied with the regular tap water for 24 hours (11 am-11 am). Food was then re-introduced with the regular tap water and 1.5% leucine solution for the control and leucine groups, respectively. The mice were allowed to feed *ad lib *for 3 hours before bloods were collected for determination of plasma glucose and insulin. Food and water intake during the 3 hour refeeding period were recorded.

### Measurement of food intake and indirect calorimetry

Daily food intake and water (or leucine solution) consumption were determined twice a week in individually-housed mice during the first two months of leucine treatment in both RCS10 and *A*^*y *^mice. Indirect calorimetry was performed in *A*^*y *^mice at the end of 4 month leucine-treatment (LabMaster, TSE Systems, Inc. Chesterfield MO). Oxygen consumption, locomotive activity, respiration exchange ratio (RER, V_CO2_/V_O2_), and food intake were measured continuously during the same 12:12 light-dark cycles. The same diet and leucine regimens were continued during indirect calorimetry. Data were collected over a 3 day period following 2 days of adaptation to the metabolic cage.

### Determination of mRNA expression

Quantitative real-time RT-PCR was used to determine mRNA expression as previously described [17]. Briefly, total RNA was isolated using RNAeasy mini-columns (Qiagen, CA), and reverse-transcribed into single-stranded cDNA using random hexamers and M-MLV Reverse Transcriptase (Invitrogen, CA). Quantitative amplification by polymerase chain reaction (PCR) was carried out using Bio-Rad iQ SYBR Green Supermix (Bio-Rad Laboratories, CA). All RT-PCR reactions were done in duplicates. Cyclophilin A mRNA level was used to normalize total RNA input. Cycle numbers at a set fluorescence threshold within the exponential amplification range (Ct) were used to calculate expression levels of the genes of interest (relative to cyclophilin A). Changes in gene expression in leucine-treated mice were expressed relatively the control values. Following gene-specific primers were used for mRNA quantification in this study: cyclophilin A, 5'-atggcactggcggcaggtcc-3' (forward), 5'-ttgccattcctggacccaaa-3' (reverse); UCP3, 5'-caagggagcggaccactcc-3' (forward) 5'-ctctctcctccagttcccatg-3' (reverse); CrAT, 5'- tgccagttgagttcctggga-3', 5'- tctgatctgaggtgagcggt-3' (reverse); CPT-1B, 5'-ggcaacagttggttccaactact-3' (forward), 5'-caggaagcttaggcatgtacgtt-3' (reverse); PPAR-alpha, 5'-tcatacatgacatggagaccttg-3' (forward), 5'-actggcagcagtggaagaatc-3' (reverse); NRF-1, 5'-ggagcacttactggagtcc-3' (forward), 5'-ctgtccgatatcctggtggt-3' (reverse); NRF-2, 5'-ggggaacagaacaggaaaca-3' (forward), 5'-ccgtaatgcacggctaagtt-3' (reverse); TBP, 5'-ggcctctcagaagcatcacta-3' (forward), 5'-gccaagccctgagcataa-3' (reverse); aP2, 5'-gacgacaggaaggtgaagagc-3' (forward), 5'-gcctttcataacacattccacc-3' (reverse); leptin, 5'-tgacaccaaaaccctcatca-3' (forward), 5'-agcccaggaatgaagtcca-3' (reverse); MCP-1, 5'-ctgaagccagctctctcttcct-3' (forward), 5'-tccttcttggggtcagcacaga-3' (reverse); TNF-alpha, 5'-ccaccacgctcttctgtcta-3' (forward), 5'-agctgctcctccacttggtg-3' (reverse); F4/80, 5'-ctttggctatgggcttccagtc-3' (forward), 5'-gcaaggaggacagagtttatcgtg-3' (reverse).

### Immunohistochemical analysis of adipose tissue

Adipose tissue was fixed in 4% paraformaldehyde for 3 days and then paraffin-embedded according to the standard procedure. Tissue sections (7 um) were stained with anti mouse F4/80 antibody (Cat # MF8000, Invitrogen, CA) and then counter-stained with hematoxylin as previously described [18]. The level of macrophage infiltration was assessed by visual examination of the positive staining under the light microscopy.

### Statistical analysis

All data were expressed as mean ± SEM. Differences between leucine-treated and control mice were assessed using t-tests (STATISTICA V6, StatSoft, Tulsa, OK). A 2-tailed p < 0.05 was considered statistically significant. Correlation analysis was used to assess relationships between HbA1C and plasma glucose and insulin levels.

## Results

### Long term leucine supplementation prevents the development of overt diabetes in RCS10 mice

**Figure 1:**
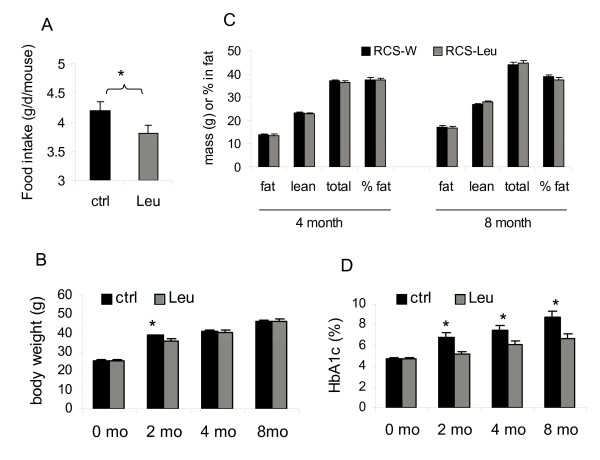
**Leucine supplementation improves glycemic control independent of energy balance in NONcNZO10/LtJ (RCS10) mice**. A: The average daily food intake measured during the first two months of leucine-treatment. B-D: Body weight (B), body composition (C), and HbA1c levels (D) in the control and leucine-treated RCS10 mice before (0 mo) and after 2, 4, and 8 months of leucine-treatment. * indicates p < 0.05, control *vs*. leucine-treated, n = 7.

Leucine supplementation was started in 8-week-old RCS10 mice and lasted for 8 months. The average water consumption measured during the first two months was not significantly different between the control (7.8 ± 0.2 ml/day) and leucine group (7.6 ± 0.1 ml/day). The average leucine intake via drinking water was 114.5 ± 15 mg/day, 1.9-fold the daily leucine intake from the chow during this period. Food intake during the first 2 months of treatment was significantly lower in leucine-treated mice (3.80 ± 0.14 g/day), relative to the control mice (4.19 ± 0.16 g/day) (p < 0.05) (Fig 1A). Weight gain during this period was also reduced in leucine-treated mice (10.1 ± 0.66 g *vs*. 13.1 ± 0.41 g, p < 0.01) (Fig 1B). However, no significant difference in body weight or adiposity was observed between the leucine-treated and control mice at the end of 4 and 8 month treatment (Fig 1B-1C).

**Figure 2:**
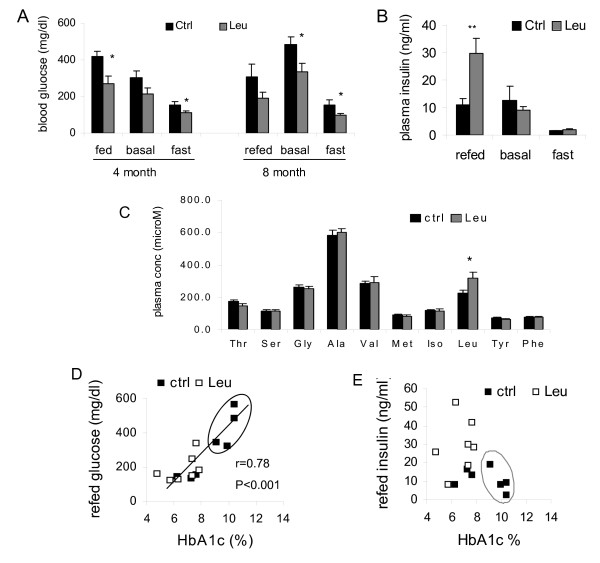
**Leucine supplementation improves glucose and insulin homeostasis and prevents overt diabetes in RCS10 mice**. A: Plasma glucose levels in the fed (or after 3 hour refed), basal (after five hour of food and leucine deprivation), and fast states in the control and leucine-treated RCS10 mice after 4 or 8 months of leucine-treatment. B: Plasma insulin levels in the refed, basal, and fast states in the control and leucine-treated mice at the end of 8 month study. C: Plasma concentrations of leucine and other amino acids after 3 hour-refeeding. D: The relationship between HbA1c levels and plasma glucose levels after the refeeding. There was a significant correlation between the two parameters (F = 29.46, p < 0.001), which fits the following equation (refed glu = -284 + 69.58x HbA1c, Standard error of estimation is 80.245). E: The relationship between HbA1c levels and plasma insulin levels after 3 hr refeeding. There was no statistically significant correlation between HbA1c levels and refed plasma insulin levels at the end of 8 month treatment. Beta cell decompensation was evident in the mice with overt diabetes (HbA1c > 9%, circled). * and ** indicate p < 0.05 and 0.01, control *vs*. leucine-treated, respectively, n = 7.

HbA1c levels were significantly lower in leucine-treated RCS10 mice relative to the control mice at the end of 2, 4, and 8 month treatment (p < 0.05 at all points) (Fig 1D). At the end of 8-month study period, more than 50% of the control mice developed overt diabetes with HbA1c levels greater than 9%, while none of the leucine-treated mice had HbA1c levels greater than 7.8%. The average HbA1c levels at 8 month were 8.68 ± 0.60% (ranging from 6.2%-10.4%) in the control groups and 6.67 ± 0.41% in the leucine group (ranging from 4.7%-7.8%). Blood glucose levels were also significantly lower or trended lower in leucine-treated RCS10 mice, compared to the control mice in all of the feeding states (Fig 2A). Basal and fast insulin levels were not significantly different at the end of 8 month-study (Fig 2B), but insulin secretion in response to refeeding was significantly more robust in leucine-treated mice than in control mice (Fig 2B). Three-hour refeeding following a 24 hr fast resulted in an average 15.8-fold increase (over the fast level) in plasma insulin levels in leucine-treated mice (ranging from 6.1 to 29.6 fold), but only 6.5-fold increase in control mice (ranging from 2.8 to 10.0 fold) (p < 0.05). The absolute plasma insulin levels after the 3 hour refeeding were also significantly higher in leucine-treated mice (29.5 ± 5.5 *vs. *11.0 ± 2.1 ng/ml, p < 0.01). Food intake during the refeeding period was not significantly different between the groups (1.23 ± 0.05 *vs*. 1.37 ± 0.15 g). Plasma leucine concentration was 38.6% (p < 0.05) higher in leucine-treated mice than in the control mice after the 3 hour refeeding; the concentrations of other amino acids examined were not significantly different between the two groups (Fig 2C). To further examine the relationship between glycemic control and beta cell function in RCS10 mice at the end of 8 month leucine treatment, HbA1c levels were related to plasma glucose and insulin levels after the 3 hour refeeding. HbA1c levels were positively correlated with the fed plasma glucose levels (r = 0.78, p < 0.001) (Fig 2D). The plotting of HbA1c levels against plasma insulin levels (Fig 2E) revealed that beta-cell decompensation was most conspicuous in the mice with HbA1c levels >9.0% (circled data points in Fig 2D and 2E), all of which were in the control group. These results suggest that the improvement in glycemic control in leucine-treated RCS10 mice may be attributable in part to the increased insulin response to feeding and decreased postprandial plasma glucose levels.

### Long term leucine supplementation improves glucose-insulin homeostasis in yellow agouti (*A*^*y*^) mice independent of weight reduction

**Figure 3:**
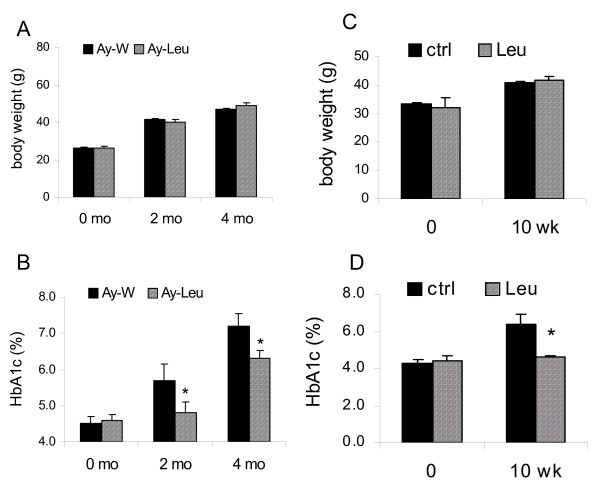
**Leucine supplementation improves glycemic control independent of energy balance in yellow agouti (*A***^***y***^**) mice**. A-B: Body weight (A) and HbA1c levels (B) of the young *A*^*y *^mice before and after 2 and 4 months of leucine treatment. Leucine supplementation and breeder chow diet were started at 2 months of age (n = 8). C-D: Body weight (C) and HbA1c levels (D) of the old *A*^*y *^mice before and after 10 weeks of leucine treatment. Leucine supplementation and breeder chow diet were started at 5 months of age (n = 6). * indicates p < 0.05, control *vs*. leucine-treated at the end of the study.

Metabolic effects of leucine supplementation were examined in two age groups of *A*^*y *^mice. In the first group, the treatment was started at 8 weeks of age and lasted for 4 months. The average food intake (3.99 ± 0.10 *vs*. 4.21 ± 0.09 g/day) and water consumption (7.3 ± 0.6 *vs*. 7.5 ± 0.7 ml/day) during the first two months of treatment were not significantly different between leucine-treated *A*^*y *^mice and their controls. Body weight was not significantly different between the control and leucine groups after 2 and 4 months of treatment (Fig 3A), nor was the body composition (data not shown). Again, HbA1c levels were significantly lower in leucine-treated mice than in control mice after 2 months (4.8 ± 0.3% *vs*. 5.7 ± 0.5%, p < 0.05) and 4 months (6.3 ± 0.2% *vs*. 7.2 ± 0.3%, p < 0.05) (Fig 3B). Effects of leucine supplementation on body weight and HbA1c levels were similar in the second group of *A*^*y *^mice (old *A*^*y*^), in which the treatment was started at 5 months of age and lasted for 10 weeks. At the end of the 10-week treatment, body weight was not significantly different between the control and leucine groups (Fig 3C), but the HbA1c level was significantly lower in leucine-treated mice, relative to the control mice (p < 0.05) (Fig 3D). While the HbA1c level rose from 4.2% to 6.4% in the control group during this period, it was virtually unchanged in the leucine-treated group (4.3% to 4.6%).

**Figure 4:**
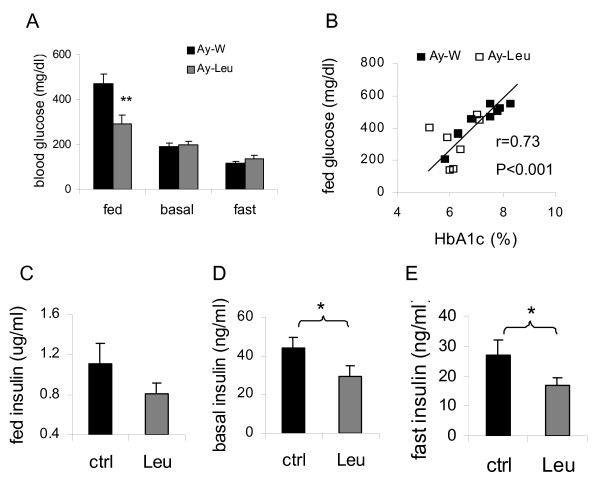
**Leucine supplementation improves glucose and insulin homeostasis in *A***^***y***^**mice**. A: Plasma glucose levels in the fed, basal and fast states after 4 months of leucine treatment. B: Positive correlation between HbA1c levels and plasma glucose levels in the fed state, which fits the following equation (fed glu = -392 + 116 × HbA1c, F = 18.1, Standard error of estimation 93.184, F = 18.1, p < 0.001). C-E: Plasma insulin levels in the fed, basal and fast states after 4 months of leucine treatment. * and ** indicate p < 0.05 and 0.01, control *vs*. leucine-treated, respectively, n = 8.

Fed plasma glucose levels were also significantly lower in leucine-treated *A*^*y *^mice than in control mice at the end of 4 month treatment (p < 0.01) (Fig 4A), and were positively correlated with the HbA1c levels in these mice (r = 0.73, p < 0.001) (Fig 4B). Although basal and fast blood glucose levels were not significantly different between the control and leucine groups, the corresponding plasma insulin levels were 38.5% and 33.9% lower, respectively, in leucine-treated mice, relative to the control mice (both p < 0.05) (Fig 4C-4D), suggesting that leucine supplementation may have improved insulin sensitivity in the basal and fast states in these mice. Plasma insulin levels in the fed state did not differ significantly between the control and leucine groups (0.80 ± 0.11 *vs*. 1.11 ± 0.21 μg ml^-1^, p = 0.16) (Fig 4E).

### Leucine supplementation increases metabolic rates and the expression of genes involved in energy metabolism in skeletal muscle

**Figure 5:**
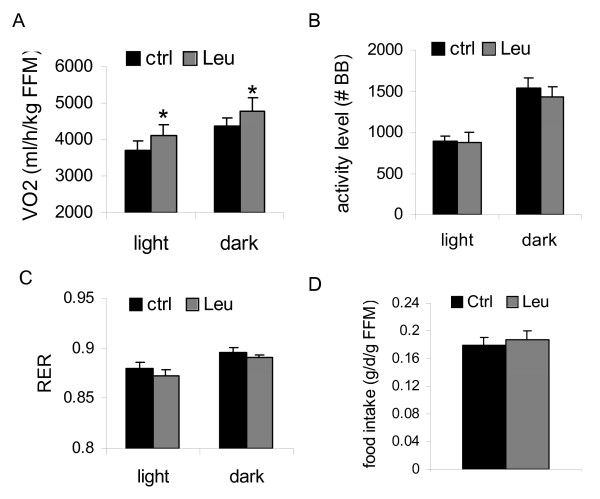
**Leucine supplementation increases the resting metabolic rates in *A***^***y***^**mice**. A-C: rates of oxygen consumption (A), locomotive activities (B) and respiratory exchange ratio (RER, V_CO2_/V_O2_) (C) in the light and dark cycles in young *A*^*y *^mice at the end of 4 month treatment. D. Food intake per unit of fat-free body mass (FFM) during the indirect calorimetric analysis. * indicates p < 0.05, control *vs*. leucine-treated, n = 8.

In order to determine the effect of long term leucine supplementation on energy metabolism, indirect calorimetry was performed in the *A*^*y *^mice at the end of 4 month leucine-treatment. Rates of oxygen consumption were ~10.7% and 8.9% higher, respectively, in the light and dark cycles in leucine-treated mice, relative to control mice (both p < 0.05) (Fig 5A). No increase in locomotive activity in leucine-treated mice was observed (Fig 5B). The respiratory exchange ratio (RER, V_CO2_/V_O2_) was slightly lower and food intake was slight higher in leucine-treated mice, compared to the control mice, although the differences were not statistically significant (Fig 5C-5D).

**Figure 6:**
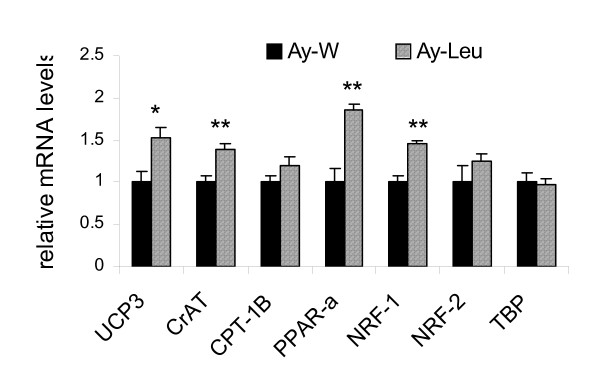
**Leucine supplementation increases skeletal muscle expression of genes involved in regulating energy metabolism in *A***^***y***^**mice**. Messenger RNA levels of uncoupling protein 3 (UCP3), carnitine acetyltransferase (CrAT), carnitine palmitoyltransferase 1B (CPT-1B), peroxisome proliferator-activated receptor alpha (PPAR-alpha), nuclear respiratory factor 1 (NRF-1), nuclear respiratory factor 2 (NRF-2), and TATA-binding protein (TBP) were determined in the soleus muscle of young *A*^*y *^mice after 4 months of treatment. The expression levels in leucine-treated mice are expressed relatively to the levels of the control mice. * and ** indicate p < 0.05 and 0.01, respectively, control *vs*. leucine-treated, n = 8.

In order to further understand the effect of leucine supplementation on energy metabolism at the molecular level, we next examined the expression level of key genes involved in fatty acid metabolism and mitochondrial function in the skeletal muscle of A^y ^mice. The mRNA levels for uncoupling protein 3 (UCP3), carnitine acetyltransferase (CrAT), peroxisome proliferators-activated receptor alpha (PPAR-alpha), and nuclear respiratory factor 1 (NRF-1) were significantly higher in the soleus muscle of leucine-treated mice than in control mice (Fig 6). The mRNA levels for carnitine palmitoyltransferase-1B (CPT-1B) and nuclear respiratory factor 2 (NRF-2) were also increased although the differences were not statistically significant. The expression level of TATA-binding protein (TBP) was unaltered. These results suggest that long term leucine treatment may increase energy expenditure by selectively stimulating the expression of a set of key genes involved in fatty acid metabolism and mitochondrial biogenesis in the skeletal muscle.

### Long term leucine-supplementation decreases adipose tissue inflammation in *A*^*y *^mice

**Figure 7:**
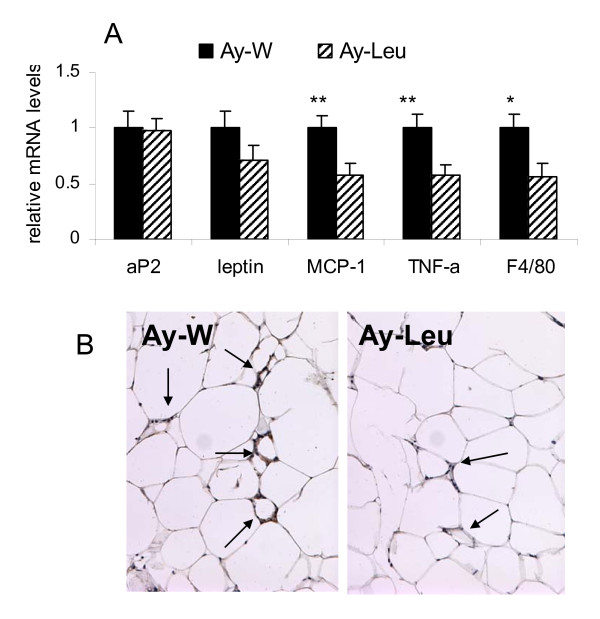
**Leucine supplementation decreases adipose tissue inflammation in A**^**y **^**mice**. A: messenger RNA levels of aP2, leptin, monocyte chemoattractant protein-1 (MCP-1), tumor necrosis factor alpha (TNF-alpha), and F4/80, a macrophage-specific marker, in the epididymal adipose tissue of leucine-treated and control young *A*^*y *^mice at the end of 4 month study period. The expression levels in leucine-treated mice are expressed relatively to the levels of the control mice. * and ** indicate p < 0.05 and 0.01, respectively, control *vs*. leucine-treated, n = 8. B. Immunohistochemical staining of F4/80 of epididymal adipose tissue of leucine-treated and control young *A*^*y *^mice. The arrows indicate the nuclei positive for F4/80 staining.

Obesity and insulin resistance have been associated with adipose tissue inflammation in both humans and rodents [18-22]. In order to assess the effect of leucine supplementation on adipose tissue inflammation, we examined the expression of several proinflammatory markers and macrophage infiltration in leucine-treated and control *A*^*y *^mice. Messenger RNA levels of monocyte chemoattractant protein-1 (MCP-1), tumor necrosis factor-alpha (TNF-alpha), and F4/80, a specific marker of macrophage, were significantly decreased in the epididymal adipose tissue of leucine-treated *A*^*y *^mice, relative to the control mice (Fig 7A). In contrast, mRNA levels of two adipocyte-specific genes, aP2 and leptin, were not significantly different between the two groups (Fig 7A). Consistent with the decreased mRNA expression of F4/80 gene, immunostaining of the epididymal adipose tissue with anti-mouse F4/80 antibody confirmed that the degree of macrophage infiltration in the epididymal adipose tissue was also decreased in leucine-treated *A*^*y *^mice, relative to the control mice (Fig 7B). These results suggest that long term leucine supplementation may attenuate adipose tissue inflammation associated with obesity.

## Discussion

We have shown in the current study that dietary leucine supplementation significantly improves glucose-insulin homeostasis in two etiologically distinct mouse models of obesity/diabetes, RCS10 and *A*^*y*^, even though the treatment has no long term effect on energy balance in these mouse models. We have further shown that the metabolic benefits of leucine supplementation in *A*^*y *^mice are associated with increased resting metabolic rates, reduced adipose tissue inflammation, and increased expression of genes involved in regulating energy metabolism and mitochondrial function in the skeletal muscle.

A novel finding of the study is that long term leucine supplementation prevents the development of full-fledged diabetes in RCS10 mice, which are prone to beta cell failure [13]. More than 50% of the control mice developed severe diabetes mellitus (HbA1c > 9%) at 10 months of age, but none of the leucine-treated mice had HbA1c higher than 7.8%. We found that leucine-treated RCS10 mice, relative to the control mice, demonstrated ~ 2-fold increase in insulin response to food challenge, suggesting that leucine supplementation may have direct effects on postprandial insulin secretion. We found that while food intake during the refeeding period was not significantly different between the two groups, leucine supplementation resulted in 38.6% increase in plasma leucine concentration in RCS10 mice at the end of 3 hour refeeding, a result similar to that observed in the DIO mouse model in our previous study [7]. Since leucine is a known insulin secretagogue [23-26], elevated postprandial plasma leucine level may be in part responsible for the more robust insulin response to feeding in RCS10 mice. Additionally, the lower plasma glucose levels in the presence of similar plasma insulin levels in leucine-treated RCS10 mice in the basal and fast states suggests that leucine supplementation may also improve hepatic insulin sensitivity in these mice, which is known to develop hepatic insulin resistance [13,14,27,28]. The increased postprandial insulin secretion and the apparently improved hepatic insulin sensitivity may both contribute to the better glycemic control and prevention of full-fledged diabetes in leucine-treated RCS10 mice.

In *A*^*y *^mice, which develop severe insulin resistance but have robust beta-cell compensations, leucine supplementation also appears to improve insulin sensitivity. Plasma insulin levels were lower in leucine-treated *A*^*y *^mice than in the control mice in all of the feeding states at the end of 4 month treatment. The lower plasma insulin levels, together with the lower HbA1c and/or plasma glucose levels are suggestive of an improvement of insulin sensitivity in these mice. Furthermore, the effects of leucine supplementation on glucose and insulin homeostasis in *A*^*y *^mice are consistent with those observed in our previous study - insulin and glucose tolerance tests have shown that leucine supplementation improves insulin sensitivity and glucose tolerance in DIO mice [7].

Leucine supplementation significantly decreased adipose tissue inflammation in *A*^*y *^mice, which could be an important mechanism for the improved glucose metabolism in these mice as adipose tissue inflammation and increased expression of pro-inflammatory cytokines have been implicated in causing insulin resistance [18,19,22,29-31]. We found that adipose tissue expression of pro-inflammatory cytokines and macrophage infiltration were both decreased in the epididymal adipose tissue of leucine-treated *A*^*y *^mice, relative to the control mice. Mechanism for the decreased adipose tissue inflammation in leucine-treated mice remains to be investigated. Activation of mTORC1 has also been shown to suppresses inflammation and lipolysis [32,33], two of the known risk factors for obesity-associated insulin resistance [29,34-36]. It is conceivable that long term leucine supplementation may lead to chronic, low grade activation of mTORC1, which in turn suppresses fatty acid release and inflammation, resulting in improved insulin sensitivity in the obese mice.

Long term leucine supplementation also has significant effects on energy metabolism in *A*^*y *^mice. Oxygen consumption was increased in both light and dark cycles in the absence of increased locomotive activity in *A*^*y *^mice, suggesting that leucine supplementation increases the resting metabolic rate in these mice. Similar increases in the resting metabolic rate are also observed in leucine-treated DIO mice as we have previously reported [7]. As in the DIO mice and RCS10 mice, plasma leucine concentration was likely elevated only in the fed state but not in the basal state in A^y ^mice. Thus, it appears that in these obese mouse models chronic dietary leucine supplementation increases energy expenditure independent of the acute effects of meal or leucine ingestion. The increases in the expression of UCP3, CrAT, PPAR-alpha, and NRF-1 in the skeletal muscle of leucine-treated *A*^*y *^mice further support this notion. Consistent with the data in *A*^*y *^mice, significant increases in the expression of the above genes as well as NRF-2 are also observed in the soleus muscle of leucine-treated DIO mice, relative to their respective controls, after 14 weeks of leucine treatment (unpublished observation). Many studies have shown that obesity and insulin resistance are commonly associated with impaired fatty acid oxidation and mitochondrial function [36-41]. Thus, improved energy metabolism and mitochondrial oxidative function could be an important mechanism for the improvements in glucose-insulin homeostasis in leucine-treated mice. The effect of leucine supplementation on the resting metabolic rate in both *A*^*y *^and DIO mice bears a striking resemblance to that of protein-rich diets [42,43], and is supportive of the postulation that leucine is a key mediator of the metabolic benefits of protein-rich diet [6,7].

The effects of chronic leucine supplementation on energy balance are complex. As discussed above, leucine supplementation increases metabolic rates in both *A*^*y *^mice and DIO mice [7]. We also found that food intake and the rate of weight gain were significantly lower in leucine-treated RCS10 mice, relative to the controls, during the first 2 months of treatment. The initial suppressive effect of leucine on food intake is not entirely unexpected as acute central administration of leucine has been shown to suppress food intake and body weight in rats [44,45]. However, despite these changes, body weight and adiposity were not significantly different in either RCS10 or *A*^*y *^mice at the end of the study period. It is possible that the lack of long term effects of leucine supplementation on energy balance in RCS10 and *A*^*y *^mice may be due in part to compensatory changes in energy intake in the later phase of treatment. As many previous studies have also shown, the regulation of energy balance in humans and rodents is redundant and compensatory changes occur when energy balance is perturbed. Furthermore, such compensatory changes appear to be strongly biased against negative energy balance (see [46,47] for review). Indeed, the increase in the metabolic rate in leucine-treated *A*^*y *^mice at the end of 4-month treatment was accompanied by a non-statistically significant increase in food intake. In our previous study, food intake was also increased in association with increased energy expenditure in leucine-treated DIO mice at the end of 14 week study period, although in this particular model the increase in food intake was apparently not sufficient to offset the large increase in energy expenditure [7].

## Conclusions

Altering dietary leucine intake has a significant impact on energy metabolism. Chronic leucine supplementation lowers HbA1c level and improves glucose and insulin homeostasis in multiple mouse models of obesity and diabetes. The metabolic benefits of leucine supplementation are associated with increased metabolic rates, improved gene expression profile in skeletal muscle, and decreased inflammation in adipose tissue, but are not necessarily dependent of weight reduction.

Dietary intervention, either alone or as part of a therapeutic regimen, is important in the prevention and management of obesity and type 2 diabetes. Although the physiology of human and mouse are clearly distinct, they nonetheless share many common derangements in insulin action and beta cell function that lead to type 2 diabetes. Thus, increasing dietary leucine intake via leucine supplementation may also prove beneficial to obese individuals susceptible to impaired glucose metabolism and type 2 diabetes. The efficacy of leucine supplementation in improving glucose-insulin homeostasis in obese humans should be investigated.

## List of Abbreviations used

RCS10: NONcNZO10/LtJ; *A*^*y*^: B6.Cg-*A*^*y*^/J; UCP3: uncoupling protein 3; (CrAT): carnitine acetyltransferase; PPAR-alpha: peroxisome proliferators-activated receptor alpha; NRF-1: nuclear respiratory factor 1; NRF-2: nuclear respiratory factor 2; CPT-1B: carnitine palmitoyltransferase 1B; TBP: TATA-binding protein; MCP-1: monocyte chemoattractant protein-1; TNF-alpha: tumor necrosis factor alpha; DIO: diet-induced obesity; BCAA: branched-chain amino acid; mTORC1: mammalian target of rapamycin complex 1.

## Competing interests

The authors declare that they have no competing interests.

## Authors' contributions

KG, JH, and YZ contributed to the acquisition and analysis of data; YY and YZ contributed to the conception and design of the study and writing the manuscript. YZ is responsible for the content of the manuscript. All authors have read and approved the final manuscript.
